# Therapeutic Benefit of Upadacitinib in Severe Post-Proctectomy (Class 4) Perianal Crohn’s Disease: A Three-Patient Case Series

**DOI:** 10.1093/ibd/izaf216

**Published:** 2025-09-24

**Authors:** Luke Nathan Hanna, Baseer Quraishi, Theo Pelly, Phillip Lung, Phil Tozer, Ailsa Hart, Serre-Yu Wong, Nick Powell

**Affiliations:** Department of Metabolism, Digestion and Reproduction, Imperial College London, London, United Kingdom; Department of Medicine, Icahn School of Medicine at Mount Sinai, New York, NY, United States; Department of Surgery, St Mark’s Hospital & Academic Institute, London, United Kingdom; Department of Radiology, St Mark’s Hospital & Academic Institute, London, United Kingdom; Department of Surgery, St Mark’s Hospital & Academic Institute, London, United Kingdom; Department of Medicine, St Mark’s Hospital & Academic Institute, London, United Kingdom; The Dr Henry D. Janowitz Division of Gastroenterology, Icahn School of Medicine at Mount Sinai, New York, NY, United States; Department of Metabolism, Digestion and Reproduction, Imperial College London, London, United Kingdom

**Keywords:** perianal Crohn’s disease, small molecules, upadacitinib, persistent perineal sinus, perineal wound, surgical complications, proctectomy outcomes, “Class 4”, perianal Crohn’s disease

## Abstract

This case series describes striking clinical and radiological responses in 3 Crohn’s disease patients with persistent perineal complications following proctectomy treated with upadacitinib. It highlights the potential of advanced therapies for this refractory inflammatory bowel disease phenotype with limited treatment options.

Key Messages **What is already known?** 

- Approximately, 30% of IBD patients undergoing proctectomy experience persistent perineal sinuses and nonhealing wounds at 1 year.
- The TOpClass system categorizes these as “Class 4” perianal Crohn’s disease.
- Class 4 disease is poorly characterized, with limited evidence on the role of advanced medical therapies.

**What is new here?** 

- We describe 3 patients with Class 4 disease who demonstrated symptom improvement and perineal healing with upadacitinib, attributed to therapy.

**How can this study help patient care?** 

- These cases highlight a possible role for advanced therapies in select, refractory Class 4 cases. Prospective studies are needed to define efficacy and inform treatment algorithms.

## Introduction

Perianal Crohn’s disease is a difficult-to-treat inflammatory bowel disease (IBD) phenotype^1^ where the last resort is proctectomy.^1^^,^^2^ Post-proctectomy, poor perineal healing is seen in approximately 30% of patients at 12 months, in the form of nonhealing wounds and persistent perineal sinuses.^3^ This group is termed in the *TOpClass Classification* as those with “Class 4” disease.^4^ The optimal management of this group is unknown; however, advanced therapies may be utilized in selected cases to reduce perineal inflammation and encourage healing.^2^ Therefore, upadacitinib, a selective Janus Kinase 1 inhibitor, may represent a therapeutic option. A recent post hoc analysis of upadacitinib studies suggested promising results for Crohn’s perianal fistula.^5^ However, evidence regarding its use in perineal disease post-proctectomy is limited. We therefore present 3 upadacitinib-treated Class 4 patients from St Mark’s Hospital (United Kingdom) and Mount Sinai (United States).

## Methods

Patients with perineal complications following proctectomy who were treated with upadacitinib were identified via screening of small-molecule prescribing records between March 2022 and June 2025. Clinical notes, imaging, and wound assessments were retrospectively reviewed. Data on patient characteristics, symptom duration, prior therapies, and upadacitinib dosing were extracted. Outcomes were assessed via clinical examination and radiology, as documented by the treating clinicians. Local institutional approvals were obtained, and all patients provided written consent for inclusion in this report. It is notable that all proctectomies were performed in mid-2022, prior to the May 2023 (United States Food and Drug Administration) and February 2023 (Medicines and Healthcare products Regulatory Agency) licensing of upadacitinib for Crohn’s disease. Consequently, all patients were naïve to upadacitinib at the time of surgery, and it was subsequently initiated for persistent post-proctectomy disease.

### Results

Key details and treatment outcomes of the cases are given in Table 1.

**Table 1.** Patient demographics, baseline disease characteristics, prior IBD therapies, and key outcomes.

Case	Demographics				Disease details		Treatment history	Outcomes on upadacitinib			
	**Age at induction (years)** ^a^	Gender	Ethnicity	Smoking status	Montreal	Perineal issue	Prior therapies:	Clinical & radiological outcome	Time to effect^b^	Follow-up period on upadacitinib	Treatment side effects
1	33	Female	White	Nonsmoker	A1, L2, B1p	Persistent sinus	Azathioprine—leukopenia Infliximab—anaphylaxis Adalimumab—loss of response Ustekinumab—loss of response Vedolizumab—stopped at time proctectomy (after 4 infusions)	Clinical: Resolved sinus drainage	2 months	19 months Therapy ongoing (30 mg once daily)	Well tolerated
								Radiological: Reduced sinus volume and enhancement	3 months^c^		
2	35	Male	Mixed-race	Ex-smoker	A2, L3, B2p	Persistent sinus	Azathioprine—leukopenia Infliximab—loss of response Ustekinumab—poor response Adalimumab—patient-reported recurrent infections	Clinical: Reduced perineal pain and sinus drainage	3 months	27 months Therapy ongoing (15 mg once daily)	Recurrent (minor) infections on 30 mg dosing
								Radiological: Reduced sinus volume and enhancement	15 months^c^		
3	27	Male	White	Nonsmoker	A2, L3, B2/3p	Nonhealing wound	Infliximab—loss of response Ustekinumab—poor response Adalimumab—loss of response Methotrexate—stopped once upadacitinib established	Clinical: Partial wound healing	2·5 months	22 months Therapy ongoing (30 mg once daily)	Well tolerated
								Radiological: Reduced wound enhancement; resolution of associated pelvic collection	2 months		

a Age at drug induction (rather than age at proctectomy, which is given in manuscript prose).

b Time to initial observed beneficial effect, based on retrospective chart review.

c Represents the time of first pelvic imaging performed after upadacitinib induction for radiological outcomes, and the time to first documented symptomatic improvement for clinical outcomes.

### Case 1

**Figure 1.**
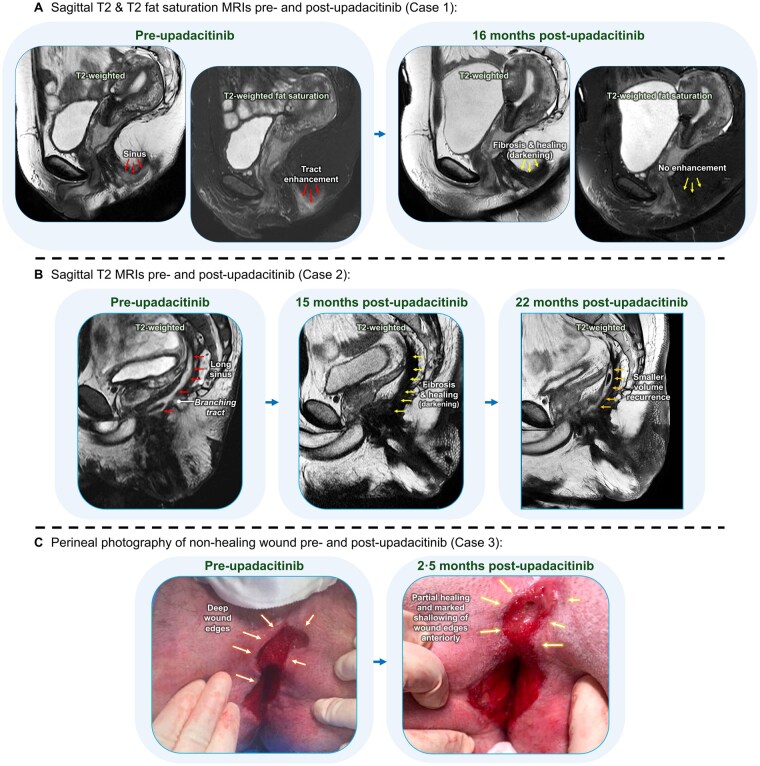
Magnetic resonance imaging (MRI) and perineal photography demonstrating perineal disease pre- and post-upadacitinib.

A 31-year-old woman with colonic and perianal Crohn’s disease underwent a completion proctectomy for persistent fistulizing disease of the rectal stump. Resection histology demonstrated granulomatous proctitis with ulceration and fissuring extending into the anal canal. At 2 months postoperatively, her perineal wound was healing well apart from a central area of shallow wound dehiscence. By 6 months, nonhealing at this site continued with new symptoms of perineal discharge. Magnetic resonance imaging (MRI) demonstrated a wide sinus at the excised left anal canal. Unfortunately, her stoma was also causing issue with mucosal inflammation, bleeding, and mucocutaneous eruptions. She was initiated on a 12-week induction of 45-mg upadacitinib, followed by 30-mg maintenance therapy. At 2-month therapy, sinus discharge had ceased. Follow-up MRIs at 3 and 16 months demonstrated sinus healing and fibrosis (Figure 1A).

### Case 2

A 34-year-old man with aggressive ileocolonic and perianal Crohn’s disease underwent proctectomy. Resection histology demonstrated rectal ulceration, granulomata, and deep fissuring into the anal canal. Postoperatively, his wound dehisced, causing severe pain and discharge. MRI at 2.5 months postoperatively revealed a long presacral sinus, with multiple branches to the natal cleft.

Despite 2 admissions for surgical drainage, symptoms persisted. He was off IBD therapy, having stopped adalimumab post-proctectomy. Due to a high-output stoma and weight loss, MR enterography was performed, showing active ileal inflammation and colitis in the defunctioned colon.

At 11 months post-proctectomy, he was initiated on a 12-week induction of 45-mg upadacitinib, followed by 30-mg maintenance therapy. At 3-month follow-up, he reported reduced perineal pain. Adjunctive surgical curettage of the sinus tracts was performed at 4 months post-induction. After 8 months of upadacitinib, he continued to report improved quality of life and reduced pain, although mild sinus discharge persisted, prompting repeat surgical drainage at 10 months. Follow-up MRI at 15 months demonstrated marked sinus healing, although subsequent imaging at 22 months showed some re-accumulation of sinus fluid (Figure 1B). Symptomatic improvement was sustained; however, recurrent infections necessitated a dose reduction of upadacitinib to 15 mg daily at 26 months of therapy.

### Case 3

A 26-year-old man with severe ileocolonic and perianal Crohn’s disease underwent a completion proctectomy for persistent anorectal disease. Proctectomy histology demonstrated ulcerating, transmural, proctitis with granulomata extending to both resection margins. His postoperative course was complicated by recurrent gluteal cleft abscesses and poor wound healing. At 14 months post-proctectomy, he was hospitalized for 3 months with extensive perineal wound breakdown, requiring serial debridement and temporary negative pressure wound therapy. Upadacitinib was initiated in hospital, with 12-week induction at 45-mg dosing. MRI following 2 months of upadacitinib demonstrated minimal wound enhancement and no evidence of ongoing pelvic collections. Healing was documented photographically (Figure 1C). He continued 30-mg maintenance dosing. At 15 months of therapy, he reported clinical stability with increasing weight and no wound discharge. At 22 months, he reported continued symptomatic improvement, with no further surgical interventions required.

## Conclusion

This series highlights a small but encouraging experience in an understudied patient population. Extrapolating from observational data in niche conditions such as Class 4 disease remains challenging without controlled studies. While spontaneous healing cannot be excluded, retrospective assessment suggested that both clinicians and patients perceived benefits attributable to upadacitinib. In refractory Class 4 patients, where histology or clinical features suggest ongoing IBD-related inflammation in the proctectomy bed or perineal tissues, this therapy could reasonably be considered within a multimodal approach, alongside surgery, wound care, topical agents, hyperbaric oxygen, and other advanced therapies.^2^^,^^4^^,^^6^^,^^7^ However, prospective studies are needed to clarify its efficacy, as with other IBD medications, in this complex setting.
